# Field-Based Calibration of Unmanned Aerial Vehicle Thermal Infrared Imagery with Temperature-Controlled References

**DOI:** 10.3390/s20247098

**Published:** 2020-12-11

**Authors:** Xiongzhe Han, J. Alex Thomasson, Vaishali Swaminathan, Tianyi Wang, Jeffrey Siegfried, Rahul Raman, Nithya Rajan, Haly Neely

**Affiliations:** 1 Department of Biosystems Engineering, College of Agriculture and Life Sciences, Kangwon National University, Chuncheon 24341, Kangwon, Korea; 2 Department of Agricultural and Biological Engineering, Mississippi State University, Starkville, MS 39759, USA; athomasson@abe.msstate.edu; 3 Department of Biological and Agricultural Engineering, Texas A&M University, College Station, TX 77843, USA; vaishaliswaminathan@tamu.edu; 4 Texas A&M AgriLife Research, Dallas, TX 75252, USA; tianyi.wang@ag.tamu.edu; 5 Department of Soil and Crop Sciences, Texas A&M University, College Station, TX 77843, USA; jeff.siegfried@tamu.edu (J.S.); rahul_11117@tamu.edu (R.R.); nrajan@tamu.edu (N.R.); 6 Department of Crop and Soil Sciences, Washington State University, Pullman, WA 99164, USA; h.neely@wsu.edu

**Keywords:** unmanned aerial vehicles, thermal remote sensing, temperature-controlled ground references, crop surface temperature, temperature calibration

## Abstract

Accurate and reliable calibration methods are required when applying unmanned aerial vehicle (UAV)-based thermal remote sensing in precision agriculture for crop stress monitoring, irrigation planning, and harvesting. The primary objective of this study was to improve the calibration accuracies of UAV-based thermal images using temperature-controlled ground references. Two temperature-controlled ground references were installed in the field to serve as high- and low-temperature references, approximately spanning the expected range of crop surface temperatures during the growing season. Our results showed that the proposed method using temperature-controlled references was able to reduce errors due to ambient conditions from 9.29 to 1.68 °C, when tested with validation panels. There was a significant improvement in crop temperature estimation from the thermal image mosaic, as the error reduced from 14.0 °C in the un-calibrated image to 1.01 °C in the calibrated image. Furthermore, a multiple linear regression model (*R*^2^ = 0.78; *p*-value < 0.001; relative RMSE = 2.42%) was established to quantify soil moisture content based on canopy surface temperature and soil type, using UAV-based thermal image data and soil electrical conductivity (ECa) data as the predictor variables.

## 1. Introduction

Remote sensing of plant temperatures has been used in breeding for the identification of traits related to disease resistance [1,2], water stress tolerance [1,3], and tolerance to other biotic and abiotic stresses [1,2,3,4]. It has also been used to support on-farm decision making in conjunction with maps of soil moisture [5], soil texture [5], and crop yield [5], as well as irrigation and nitrogen statuses [6].

Most of the plant canopy and soil temperature measurements currently employed use contact probes or thermometers [7,8,9] and non-contact type hand-held infrared thermometers [10,11,12,13]. Brinkhoff et al. [7] presented the use of multisensory capacitance probes for the simultaneous monitoring of ponded water level, soil moisture, and temperature profile for soil water content monitoring and irrigation scheduling. The contact probes showed good performance in terms of cost, accuracy, and reliability; however, it is time-consuming and labor-intensive and thus excessively costly if it needs to be repeated multiple times in a season. Wang et al. [13] proposed using an inexpensive infrared optical sensor for reliable and precise canopy temperature measurements in a large rice field; however, errors due to environmental effects were not considered in the study, and because these non-contact sensors were also non-imaging sensors, they had low spatial detail and did not provide much information about variability across the field. It is possible to extract field variability and crop status information via satellite and aerial thermal remote sensing [14,15,16,17] by analyzing the spatial temperature patterns from images. However, thermal image data acquired with unmanned aerial vehicles (UAVs) may be more effective for agronomic applications because of their potentially higher temporal and spatial resolutions [18,19,20,21].

UAV-based thermal remote sensing of agricultural fields has been tested for a wide range of applications thus far, including irrigation scheduling [20], plant disease detection [21], water status assessment [22], fruit or crop maturity evaluation [23,24], bruise detection in fruits and vegetables [25,26], and other applications involving agricultural decision making. Möller et al. [27] demonstrated that fusing thermal and visible images could improve the accuracy of physiological feature measurements such as stem water potential and stomatal conductance in vineyards. However, thermal imaging has remained underexploited for high-throughput phenotyping [28,29,30]. Further, the temperature measurements are error-prone, even though thermal camera manufacturers provide software applications to perform the necessary radiometric calibrations (e.g., IR-Flash by ICI), and precise adjustments for atmospheric errors cannot be performed. Therefore, correcting for atmospheric effects in UAV imagery is very important, yet remains a challenging process [31,32].

For more accurate temperature estimation and removal of atmospheric errors when using thermal cameras, calibrations must be performed based on black-body [33,34,35] or known ground references [36,37,38]. The calibrations of laboratory instruments involve using the instruments to measure reference objects in the same manner as the experimental samples. Then, the instrument responses are adjusted electronically to provide appropriate outputs, or the software used to process the instrument outputs are adjusted so that data from the instrument are corrected based on calibration parameters. Doing so corrects for the effects of sensor errors and atmospheric distortions. Ribeiro-Gomes et al. [39] proposed a calibration method for uncooled thermal cameras based on a neural network with the sensor temperature and digital response of each pixel as the input data; when calibrating with this methodology, the measurement error was reduced from approximately 4.0 °C to 1.5 °C. Jensen et al. [40] demonstrated two methods to calibrate thermal imagery and model external disturbances affecting camera accuracy. One method involved a ground-based thermal camera mounted on a long boom to capture high-resolution samples of the field temperature, which were then used to calibrate the aerial mosaic. The other method involved using cool and warm pools of water that were actively controlled by pumping into a heat-transfer reservoir, which was then used for calibrating the aerial thermal images. The pools-based method had the best residual norm of 1.6, closely followed by the ground-based thermal camera method with a residual norm of 1.96. However, it was difficult to choose the more accurate method between these two without sample temperature measurements from the field for ground truth. Moreover, the demonstrated methods are cumbersome and most likely impractical on farms if this process must occur multiple times during a growing season. Kelly et al. [41] used a simple empirical line calibration method based on three ground calibration panels composed of wooden frames with different colors to produce a wide temperature range; this method allowed converting digital numbers from a camera to temperature values for images captured during UAV flights, with an accuracy of ±5 °C and substantial improvements over temperature estimates within a ±20 °C error range, in the case of thermal images obtained without effective radiometric calibrations. However, the uncontrolled temperature calibration panels were not able to span the expected range of crop surface temperatures during the summer growing season and did not achieve significant improvements in temperature calibration over the UAV-based thermal data to provide accurate temperature measurements.

While past studies used various calibration methods, such as collecting ground-level sample data with IR thermometers and probes before or after flights, there is a dearth of research on using temperature-controlled ground references for more accurate temperature estimates from thermal images. Jensen et al. [40] and Kelly et al. [41] focused on temperature calibration methods for aerial thermal images, and their results illustrated that calibration systems based on ground references showed improved and potentially acceptable accuracies and performances at some level. However, there is still room to improve the ground-temperature reference designs for robustness and stability, as well as a need to evaluate the calibration accuracies with these methods.

It is clear that the accuracies of thermal image data acquired from UAVs could greatly benefit from thermal references on the ground. However, the methods used in prior research were not adapted for farming operations or even large research programs, as they lacked stability under dynamically varying temperature conditions. Thus, the goal of the present research is to demonstrate the possibility of calibrating UAV-based thermal images with practical implementation of electronics-based temperature-controlled ground references to improve the accuracy compared to existing methods [39,40,41]. The specific objectives of this study were (1) to develop ground temperature references with appropriate dynamic ranges for plant temperature measurements in the field for precise calibration of UAV thermal images; (2) to evaluate the benefits of using the proposed ground temperature references for calibrating surface temperatures in terms of accuracy improvements; and (3) to determine the relationships between UAV-based canopy temperature estimates and soil characteristics to demonstrate the importance of accurate canopy-temperature data, since soil moisture content is a primary factor affecting farm productivity owing to its influence on plant growth, microbial activity, irrigation management, and water-use efficiency [42,43].

## 2. Materials and Methods

### 2.1. Equipment

#### 2.1.1. UAV Platform and Camera

**Figure 1:**
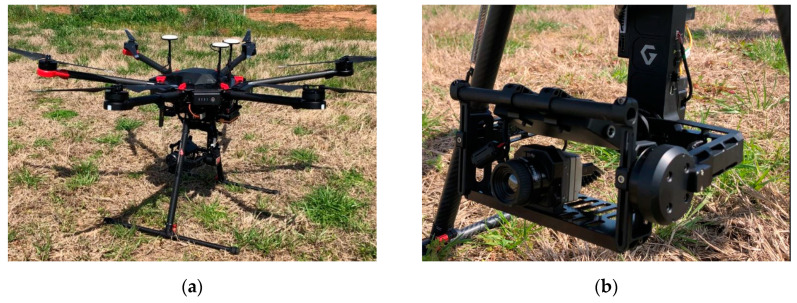
Unmanned aerial vehicle, (**a**) DJI Matrice 600 Pro and (**b**) ICI thermal camera.

Thermal images were acquired using a rotary-wing UAV (Matrice 600 Pro, DJI, Guangdong, China; Figure 1), which was autonomously controlled along a predefined flight path with Pix4Dcapture mission planning software (Pix4D SA, Lausanne, Switzerland). The UAV has a maximum payload of 6 kg and was equipped with a thermal camera (ICI 8640 P-series, Infrared Cameras Inc., Beaumont, TX, USA) mounted on a three-axis gimbal to stabilize the images acquired during flight. The thermal camera produced 640 × 512 resolution images and was pre-calibrated by the manufacturer, and the temperature data were embedded in Celsius and Kelvin units in each pixel as calibrated temperature values of 16-bit and 32-bit tiff images, respectively. 

#### 2.1.2. Temperature-Controlled References

**Figure 2:**
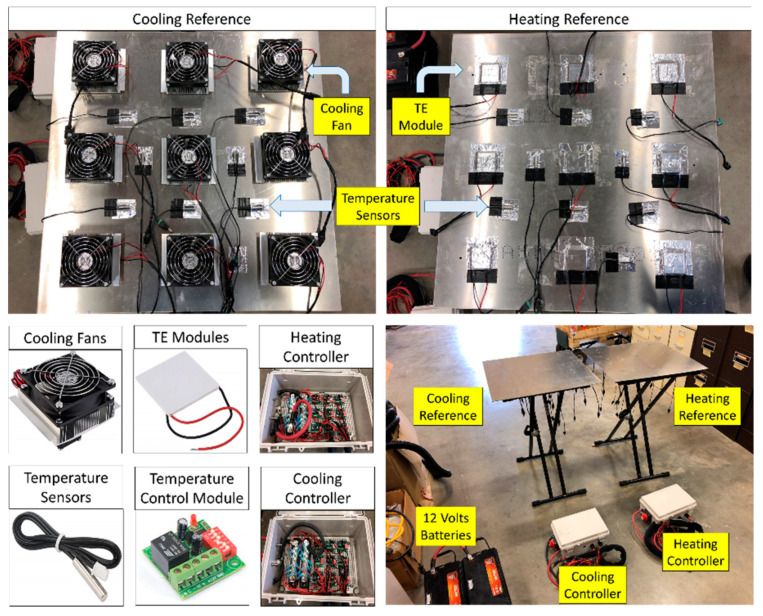
Individual components of the controlled high- and low-temperature references.

**Figure 3:**
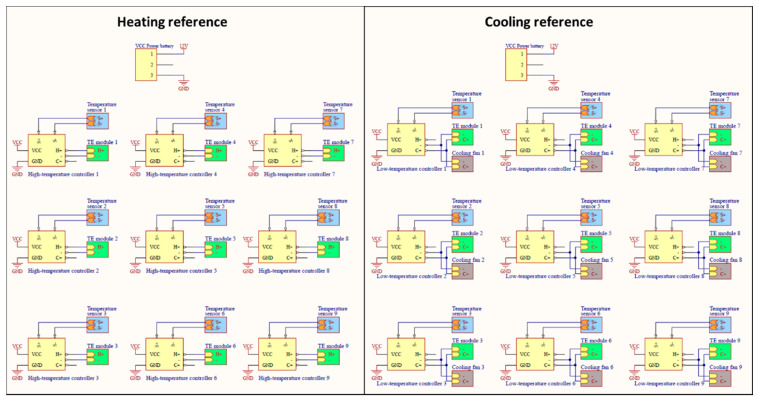
Schematic diagram of the circuit system of the temperature-controlled references.

**Figure 4:**
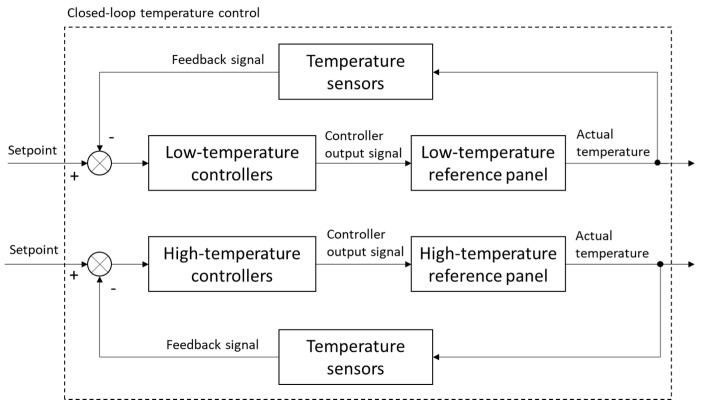
The architecture of the closed-loop temperature control method, consisting of a low-temperature reference control and a high-temperature reference control.

Both the temperature-controlled reference measurements were based on a 61 × 61 cm square aluminum plate. These were chosen to provide at least 40 pixels in the aerial thermal images when the camera was flown 40 m above the ground level (AGL). One reference served as a high-temperature reference and was equipped with temperature sensors, thermoelectric (TE) modules, and an integrated heating controller. The other reference served as a low-temperature reference and was equipped with temperature sensors, cooling fans, and an integrated cooling controller (Figure 2). Nine TE modules (Watronix, CA, USA) and nine temperature sensors (Droking, Guangdong, China) were uniformly distributed across both the references. To dissipate heat efficiently in the low-temperature reference panel, cooling fans (ARCTIC, Brunswick, Germany) were mounted atop each TE module. The circuit system comprised a heating reference circuit and a cooling reference circuit; its schematic diagram is shown in Figure 3. Nine high- and nine low-temperature microcontroller modules (Droking, Guangdong, China) were connected in parallel for both the high- and low-temperature references. The temperature sensors that produce continuous analog output signals were directly connected to analog channels of the microcontroller modules; the TE modules and cooling fans that connect to digital channels of the microcontroller modules were actuated with digital signal processing. The surface temperatures of both references were controlled with the nine microcontroller modules powered by a 12 V, 310 Ah rechargeable battery, based on a closed-loop temperature control method to accurately control and maintain temperature during the process (Figure 4). The control method, which is an efficient method for various industrial processes, contains a feedback loop in which the control system receives analog signals measured from the corresponding temperature sensors and develops a response to achieve temperature stability. The reference temperatures in the low and high reference controllers were set to 20 and 50 °C, respectively, based on the capacity of the battery to span the expected range of crop surface temperatures during the summer growing season.

### 2.2. Field Methods

#### 2.2.1. Test Site

**Figure 5:**
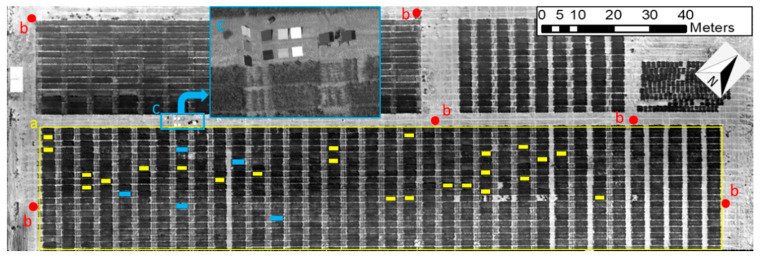
Experiment field at Texas A&M AgriLife research farm. (**a**) Delineations of the wheat field. (**b**) Ground control points. (**c**) Details of the test equipment on the ground, including temperature-controlled references and validation panels.

Experiments were conducted in a winter wheat field at the Texas A&M AgriLife research farm (headquarters at latitude 30.549635 N, longitude 96.436821 W in WGS-84 coordinate system) near College Station, TX, USA. The total field area covered during the experimental flight was 6000 m^2^ and included 300 wheat plots arranged in 20 rows of 5 × 2 m each (Figure 5). The row spacing for each plot was 0.18 m. A total of fifty uniform varietal trial genotypes (hard red and soft red winter wheat) with six replicates were planted in a randomized complete block design across the field on 30 October 2018, and six ground-control points (GCPs) were distributed across the field to increase the geographical accuracy of the temperature map. The ambient temperature at the time of data collection on 6 March 2019 was approximately 15.6 °C.

#### 2.2.2. Flight Procedures

The UAV flight mission combined with a 12.5 mm focal length of the thermal camera was carried out at solar noon with no cloud cover. The thermal camera system was powered on in advance for more than half an hour for warming up before UAV-based thermal image collection to produce stable temperature readings. The UAV was flown at 40 m AGL with a flight speed of 2.5 m/s to provide a ground resolution of approximately 5 cm/pixel and involved a grid pattern with back-and-forth flight lines that had the UAV pass three times over the test equipment at the side of the field. Image overlap was set to 80% in both the forward and sideward directions, and the camera was triggered at a predefined trigger interval of 2 s with flight speed calculated to achieve the desired overlap.

#### 2.2.3. Temperature Validation Tests

**Figure 6:**
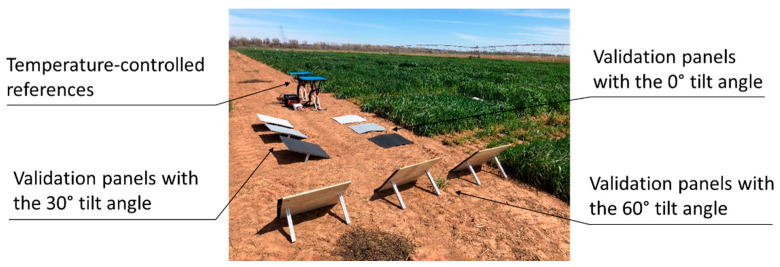
Temperature-controlled references, three sets of three-colored square validation panels placed on the ground at different tilt angles, and ground-truth temperature measurements.

To investigate the benefit of calibrating the thermal image mosaic with field-based references, the temperature-controlled references, which were supported on metal stands, were placed on a flat road next to the test field (Figure 6). The temperature-controlled references were powered on 6 min before the UAV flight to achieve stable surface temperatures, and continuous power supply was maintained over the entire duration of the flight. Validation panels were installed next to the temperature references for comparisons between the ground-measured temperatures of the panels and those obtained from the temperature-corrected thermal image mosaics. These validation panels consisted of three sets of three-colored (light gray, 50% reflectance; medium gray, 25% reflectance; dark gray, 50% reflectance) square panels of dimensions 61 × 61 cm placed at three tilt angles (0, 30°, SE; 60°, NE) to impose temperature variations due to relative sun angles. Temperature calibration of the thermal image mosaic was implemented by (1) extracting the median pixel values from the high- and low-temperature references, (2) creating a linear equation to relate the pixel values to known temperatures of the references, and (3) calibrating all other pixels in the image mosaic with this equation.

The effectiveness of the proposed temperature calibration method was evaluated by comparing the corrected and uncorrected thermal image mosaics with temperatures measured using a Handheld Infrared Radiometer (MI-220, Apogee Instruments Inc., Logan, UT, USA). The measurement targets included the aforementioned validation panels and the five randomly selected wheat plots from specific regions of interests (ROIs, solid blue blocks in Figure 5) in the test field. Temperature measurements of the five different sample locations in each plot were averaged immediately after UAV flight at solar noon on a clear sunny day and later compared to the corresponding sample averages for the calibrated and un-calibrated thermal image mosaics. The mean, root-mean-squared error (RMSE; Equation (1)), relative RMSE (Equation (2)), and RMSE improvement (Equation (3)) were calculated for each case.

(1) $$RMSE=\sqrt{\frac{\sum_{i=1}^{n}{\left(y_{i}-{\hat{y}}_{i}\right)}^{2}}{n-1}}$$

(2) $$Relative\;RMSE=\frac{RMSE}{{\bar{y}}_{i}}\times 100\%$$

(3) $$RMSE\;Improvement=\frac{\left|RMSE_{uncalibrated}-RMSE_{calibrated}\right|}{RMSE_{uncalibrated}}\times 100\%$$

where $y_{i}$ and ${\hat{y}}_{i}$ are the measured and predicted data, respectively, ${\bar{y}}_{i}$ is the mean of the measured value, and n is the total number of samples.

### 2.3. Image Data Processing

#### 2.3.1. Ortho-Mosaicking Process

Prior to image mosaicking, radiometric calibrations of the individual thermal images were performed with the IR-Flash software provided by the camera manufacturer. The software used an internal factor to convert the digital values in JPEG format images to radiometric temperature values (°C) stored in the 16-bit tiff format. The geotagging of the thermal images was completed through matching timestamps of individual images along with that of the GPS information. The georeferenced 2D ortho-mosaic of the thermal images was then obtained from 3D point clouds generated through structure-from-motion with Pix4Dmapper (Pix4D SA, Lausanne, Switzerland) software. Georeferencing was performed for the image mosaic with GCP coordinates surveyed using a real-time kinematic (RTK) global positioning system (GPS) receiver with <2 cm accuracy.

#### 2.3.2. Temperature Calibration

As previously noted in the introduction, radiometric calibrations conducted with the IR-Flash software do not comprehensively account for the influences of air temperature, ambient humidity, target object emissivity, and temperatures of the surrounding objects, which can induce errors in aerial temperature measurements of the vegetation [44,45,46]. Thus, the temperature-controlled references were deployed to remove the errors induced by ambient conditions to render the radiometric temperature values in the thermal mosaic as close as possible to the actual surface temperatures on the ground.

#### 2.3.3. Correlation Analysis between Canopy Temperature and Soil Properties

Twenty-four representative plots were selected from the mosaicked field image to study the correlation between canopy surface temperature of the winter wheat genotypes planted and properties of the underlying soil. ENVI 5.1 software (Harris Geospatial Solutions, Boulder, CO, USA) was used to obtain data from specific ROIs (solid yellow blocks in Figure 5). A 15 cm buffer was used to exclude areas along the perimeters of the wheat plots in the ROIs to avoid edge effects between the genotypes that might be caused by foliage encroachments from adjacent plots. Soil volumetric moisture contents were collected in the selected 24 plots using a ML3 Theta probe and HH2 moisture meter (Delta-T devices, Burwell, Cambridge, UK) immediately after the UAV flight. One measurement per plot at 6 cm soil depth was collected; it is commonly accepted that soil moisture in the top horizons of the soil is most important for many crops and significantly affected by temperature, especially when the plots have plants with fibrous root systems at the early growth stage and less vegetation cover or soil surface exposed to incident solar radiation [47,48,49]. The ML3 Theta probe was provided with calibration for mineral and organic soil that can convert sensor output for soil moisture content ($m^{3}/m^{3}$) when connected with the HH2 moisture meter. Additionally, an EM38-MK2 electromagnetic induction meter (Geonics Limited, Mississauga, ON, Canada), which is a proximal non-contact electromagnetic induction sensor, was used to measure the apparent soil electrical conductivity (ECa in units of mS/m) for in-field soil variability assessment two months after germination. To prevent any drift in data, pre-noon time of a partly cloudy day was selected for measurement. Finally, the ability of the UAV-based thermal remote sensing approach to provide information on spatial variations in soil properties across the field was investigated. A multiple linear regression model was developed to estimate the soil moisture content based on canopy surface temperature from the UAV thermal image and soil ECa from the EM38.

## 3. Results and Discussion

### 3.1. Temperature-Controlled References

**Figure 7:**
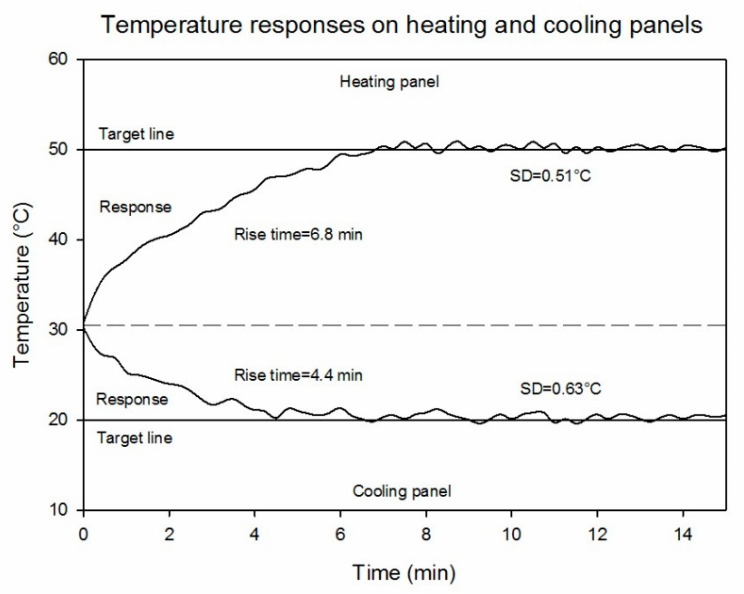
Temperature responses and stabilities of the temperature-controlled references.

Figure 7 shows the observed changes in the surface temperatures of the high and low references from the time of placement in the field to the end of the UAV flight. The high-temperature reference required at least 6.8 min to warm-up and maintain a stable temperature, while the time required for the low-temperature reference was approximately 4.4 min, which is shorter because of the cool weather during the experiment. After temperature stabilization, low standard deviations from the set values were observed (0.51 and 0.63 °C for the high- and low-temperature references, respectively). The high and low ground reference temperatures spanned the range of temperatures present in the field at the time of data collection. The experiment was conducted during winter when the ambient temperature was low, so the observed canopy temperatures were closer to the low reference temperature (20 °C). While the reference temperatures used in this study were chosen for summer conditions, they can be modified in the future to improve calibration accuracy during colder weather conditions. Therefore, the reference high- and low-temperatures should be set based on the expected maximum deviation from ambient temperature during the experiment day, as canopy temperatures measured with an infrared thermometer are highly related to ambient temperature in both stressed and non-stressed crops [50,51].

### 3.2. Temperature Accuracy Assessments

**Figure 8:**
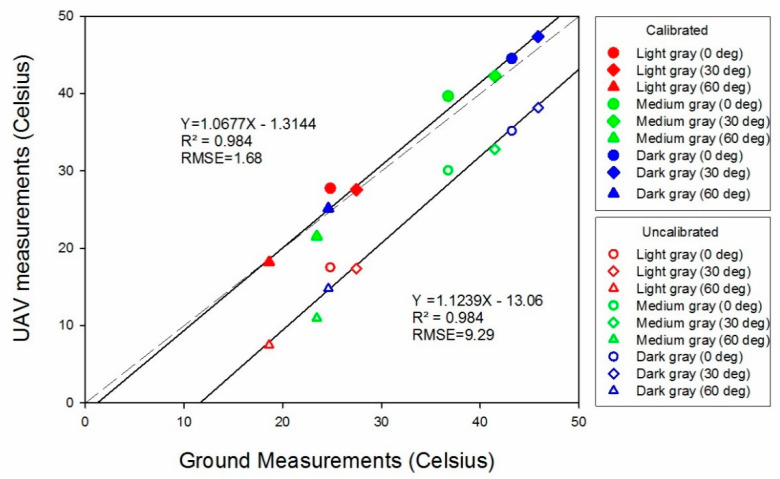
Accuracy assessment results of the unmanned aerial vehicle (UAV)-based temperature estimates based on the temperature-controlled references.

UAV thermal-image-based temperature estimates from both the un-calibrated and calibrated mosaics had strong linear relationships ($R^{2}$ = 0.98) with ground measurements from the validation panels (Figure 8). Calibrating the data greatly reduced the RMSE values from 9.29 to 1.68 °C. Thus, the trend line from the calibrated data was much closer to the 1:1 ground-truth line (dotted line) than the un-calibrated trend line, as the IR transmission losses (scattering and diffusion) due to ambient and atmospheric effects were mitigated in the calibration process. Therefore, the proposed calibration method, based on the temperature-controlled references in the field, shows promise for greatly reducing the inherent errors of surface temperature data from aerial thermal images. As darker objects are known to absorb more thermal energy, the surface temperatures of the dark gray validation panels were highest, followed by those of the medium and light gray panels. Further, temperature values obtained from the panels with 60° tilt were the lowest among panels of the same color but with lesser tilts. However, the temperature values obtained from the panels with 30° tilt were higher than those from the 0° tilt. A likely reason for the low temperatures from panels with 60° tilt is that a lesser surface area was exposed to solar energy at the given solar angle. Furthermore, since the sun was not at the exact solar noon position at the time of the UAV flight, the panels with 30° tilt had relatively more surface area perpendicularly intercepting the radiation than panels at 0 or 60° tilt. These observations emphasize the effects of leaf angles on leaf temperatures; for example, leaves with vertical orientations are found to have lower surface temperatures [52,53]. Overall, the results of this test indicate that the proposed calibration method with temperature-controlled references can greatly improve surface temperature estimation, while caution should be exercised as to the orientation of the ground objects, such as individual leaf surfaces, because leaf angles and orientations have strong effects on temperature measurements.

The mean values of the ground-truth temperature measurements from the wheat plots were between 15.24 °C and 16.16 °C (Table 1). The un-calibrated thermal images provided inaccurate estimates, with mean values ranging from 1.52 to 3.56 °C, and thereby large RMSE values. In general, un-calibrated crop temperature estimates were unreliable, as the optimal leaf-temperature range for most plants is from 15 to 36 °C under normal atmospheric concentrations of ${\mathrm{CO}}_{2}$ [54,55]. In contrast, the means of the estimates from the calibrated thermal images ranged from 15.28 to 17.02 °C and were thus closer to the ground-truth measurements. Overall, the calibrations reduced the ranges of RMSEs for plant temperature estimations from 12.6 to 14.0 °C and for un-calibrated data to 0.41 to 1.01 °C. As mentioned previously, Kelly et al. [41] calibrated temperatures with an accuracy range of ±5 °C, up to ±20 °C for the influence of temperature estimates under changing ambient conditions. The calibration results from using the temperature-controlled references demonstrated that it is a more stable and repeatable calibration method for correcting atmospheric noises in UAV-based thermal remote sensing. From a production-agriculture point of view, this improvement in temperature accuracy of thermal remote sensing has important implications for on-farm decision making, including irrigation scheduling [56,57], plant disease detection [58,59], soil property mapping [60,61], and yield estimation [62,63]. Since the temperatures estimated by thermal infrared imagery could be significantly different at different UAV flight heights [64], further research is needed to compare the performances of crop temperature estimates at different flight heights based on the temperature-controlled references.

### 3.3. Correlation Analysis between Canopy Surface Temperature and Soil Properties

**Figure 9:**
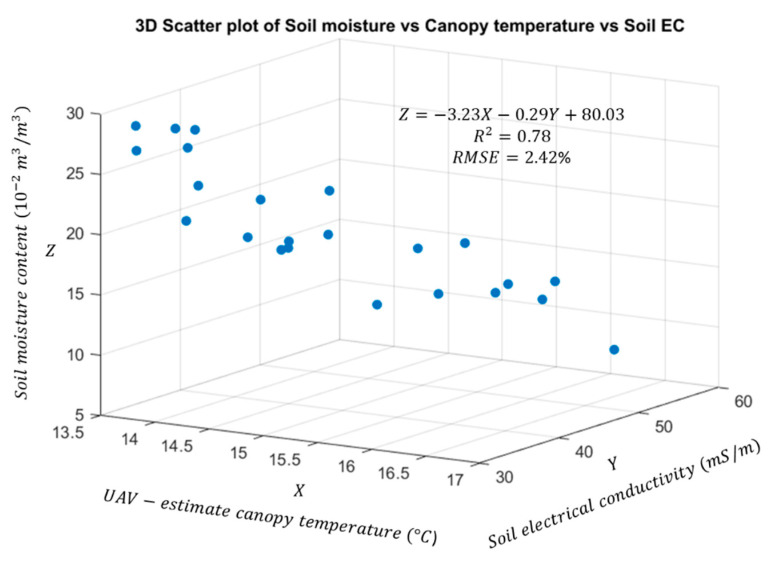
Correlation analysis between canopy temperature and soil properties (moisture content and soil ECa) across all 24 experimental plots.

Multiple linear regression between the predictor variables (UAV-based canopy temperature and soil ECa) and soil moisture produced a good fit ($R^{2}$ = 0.78; *p*-value < 0.001), suggesting good ability to estimate soil moisture content in the wheat field under study (Figure 9). A small amount of variation in the canopy temperature (13.8 to 16.7 °C) existed across the field because the crops grew under well-watered conditions, indicating that the $R^{2}$ value could be improved if the range of canopy temperatures across the field was greater, as may be encountered in non-irrigated conditions. Moreover, the multiple linear regression equation suggests that soil moisture content was negatively correlated with both canopy temperature and soil ECa, a finding that is consistent with results from other studies [65,66,67,68]. It was expected that a practical method for producing accurate canopy-temperature data across a field, which could be used for irrigation scheduling during the growing season, would enable estimating the soil moisture content with an acceptable level of accuracy (relative RMSE = 2.42%), if soil ECa data are practically available in farmland areas based on the proposed method of field-based calibration of UAV thermal images. It is notable that the canopy surface temperatures obtained from the UAV measurements were close to but lower than the low reference temperature of 20 °C, which was the lowest achievable temperature on the temperature-controlled references in the outdoor environment. Thus, further research must be conducted to improve the correlation and validate linearity between canopy surface temperature and soil properties by modifying the low-temperature reference to span the range of temperatures present in the field, for both summer and winter conditions.

## 4. Conclusions

A ground-based system of temperature-controlled references was developed to improve the temperature calibration accuracy of UAV-based thermal images obtained from 40 m AGL. The closed-loop temperature control method, applied to both the low- and high-temperature references, was able to provide stable reference temperatures for the entire duration of the UAV flight. The standard deviations from the high- and low-temperature setpoints were 0.51 and 0.63 °C, respectively, after temperature stabilization. The RMSE values were reduced after calibration from a maximum of 14 to 0.82 °C, suggesting a 94% improvement in temperature estimation from thermal image mosaics. Overall, the results of this study indicate that the proposed thermal calibration method using temperature-controlled references can provide reasonable accuracy and precision for canopy temperature estimation. Furthermore, a multiple linear regression model showed that UAV-based canopy temperature estimates along with soil ECa data can be used to estimate soil moisture content with an acceptable level of accuracy ($R^{2}$ = 0.78; *p*-value < 0.001; relative RMSE = 2.42%). In the future, the proposed temperature-controlled references should be evaluated to determine whether images captured from different flight heights show the calibration method to be repeatable when at different heights with different wind and environmental conditions [69]. Furthermore, since the current reference temperature range was designed to cover canopy temperatures during summer conditions, the power supply and method of setting reference temperatures, particularly with the low-temperature reference, need modification so as to span the range of temperatures present in the field for winter conditions, which consequently should improve the performance of the multiple linear regression model.

## Figures and Tables

**Table 1:** Temperature errors between the un-calibrated and calibrated data with temperature-controlled references.

Site	Item *	Mean (°C)	RMSE (°C)	Relative RMSE (%)	Improvement (%)
	GTT	16.16	N/A	N/A	
Location 1	UCT	3.56	12.60	78.0	92.0
	CCT	17.02	1.01	6.30	
	GTT	15.56	N/A	N/A	
Location 2	UCT	2.73	12.83	82.50	94.2
	CCT	16.31	0.75	4.80	
	GTT	15.80	N/A	N/A	
Location 3	UCT	2.12	13.68	86.60	97.7
	CCT	15.78	0.32	2.00	
	GTT	16.28	N/A	N/A	
Location 4	UCT	2.28	14.00	86.00	94.0
	CCT	15.93	0.82	5.20	
	GTT	15.24	N/A	N/A	
Location 5	UCT	1.52	13.72	90.00	97.0
	CCT	15.28	0.41	2.70	

* GTT: ground-truth temperature, UCT: un-calibrated crop temperature, CCT: calibrated crop temperature.
